# Spatiotemporal Distribution and Population Structure of *Monokalliapseudes schubarti* (Tanaidacea: Kalliapseudidae) in an Estuary in Southern Brazil

**DOI:** 10.1155/2013/363187

**Published:** 2013-11-02

**Authors:** Felipe Freitas-Júnior, Martin Lindsey Christoffersen, Joafrâncio Pereira de Araújo, Joaquim Olinto Branco

**Affiliations:** ^1^Centro de Ciências Tecnológicas da Terra e do Mar (CTTMar), Universidade Vale do Itajaí (UNIVALI), CP 360, 88302-202 Itajaí, SC, Brazil; ^2^Departamento de Sistemática e Ecologia, Universidade Federal da Paraíba (UFPB), 58059-900 João Pessoa, PB, Brazil

## Abstract

*Monokalliapseudes schubarti* is an endemic tanaidacean microcrustacean from southeastern Brazil to Uruguay inhabiting low energy estuaries. Saco da Fazenda is located in the estuary of the Itajaí-Açú River, state of Santa Catarina, Brazil. It is exposed to strong anthropic impact and receives intensive flows of domestic wastewater, solid residues, and drainage activities. Specimens of *M. schubarti* were collected monthly, in the intertidal and subtidal regions of Saco da Fazenda, in four stations defined as a function of the physiography of the environment during the period of July 2003 to June 2004. Fecundity values were high, with continuous reproductive activity during the whole period of study. The greatest population densities were observed in the intertidal region, where they are nevertheless intensely consumed by birds, swimming crabs, and fish. This species represents a fundamental link in the food chain of Saco da Fazenda, transferring energy from the detritus level to higher trophic levels. Habitat disturbance and high organic matter may represent factors controlling the distribution of populations of *M. schubarti*. For this reason, the species may be used to monitor anthropic effects in estuarine areas.

## 1. Introduction

*Monokalliapseudes schubarti* [1] is a tanaidacean microcrustacean of no known economic importance but with a relevant ecological role in estuarine environments.

It is found in the region located between Cabo Frio, Rio de Janeiro, and La Plata River, Uruguay [2, 3]. It inhabits low energy estuaries, where individuals construct “U-” shaped tubes reaching a depth of up to 15 cm [1, 4, 5]. It has been frequently cited in the literature as *Kalliapseudes schubarti* but is now included in the monotypic genus *Monokalliapseudes*, since Gutu [6] elevated the subgenus to generic rank. There are 39 species in the primarily circumtropical family Kalliapseudidae [7], which was inferred to be monophyletic on the basis of three molecular loci [8]. Members of this family, in addition to being deposit feeding as other tanaidaceans, are thought to be filter feeders based on the rows of long plumose setae on their chelipeds [9, 10]. F. P. P. Leite and P. E. P. Leite [11] provided evidence that *Kalliapseudes gianucai*, described by Bacescu [12] and known only from the type locality in the state of Rio Grande do Sul, Brazil, represents a dimorphic male stage and is thus a synonym of *M. schubarti*.

*Monokalliapseudes schubarti* displays pronounced spatial and seasonal variations in population densities having a rapid development, continuous reproductive activity, high recruitment potential, and mass mortalities, being considered an *r*-strategist [5, 13–17]. This species is important in community structuring [18] having a relevant function in the feeding of fish, crustaceans, and aquatic birds [4, 13, 19, 20]. It was found to represent a dominant species in the faunal community of Lagoa dos Patos, Rio Grande do Sul [21–24], Gamboa do Perequê, Paraná [14], Paranaguá Bay, Paraná [25, 26], and Araçá beach, São Paulo [13]. Such abundant, opportunistic, community structuring, persistent and resilient species have been found to be excellent biotic indicators and have been increasingly used as such [18, 20, 27–29].

In south and southeastern Brazil, *M. schubarti* has been studied in tidal flats [26], fine-grained beaches of low slope and high organic matter [13, 15], estuaries [16], and coastal lagoons [17].

In the state of Santa Catarina, about halfway along its total geographic range, *M. schubarti* was found to be very abundant in the estuarine area of Saco da Fazenda [30]. This paper reports the spatiotemporal distribution, population structure, and reproduction of *M. schubarti* in this environment.

## 2. Material and Methods

### 2.1. Study Area

Saco da Fazenda is located in the estuary of the Itajaí-Açú River, between 26°53′33′′–26°55′06′′ S and 48°38′30′′–48°39′14′′ W, state of Santa Catarina, Brazil. It is characterized as a shallow artificial estuary [31] because of the construction of piers to rectify and stabilize the canal of the Itajaí-Açú River. These piers have isolated a previous affluent of this river [32], and water exchange became restricted. The total surface area of Saco da Fazenda is about 6.3 × 10^5^ m^2^ [31]. The substrate is siliceous argillic and maximum depth is 2.0 m, except in the river canals which attain 9.0 m depth. Tidal variations are not higher than 1.4 m. Mean annual rainfalls range from 1250 to 1500 mm [33].

The ecosystem of Saco da Fazenda, despite its exposure to a strong anthropic impact and to an intensive discharge of domestic wastewater, solid residues [33] and drainage activities [32], supports a high diversity of animals and serves as a natural nursery for several species of crustaceans, fish, and birds, which may use *M. schubarti* as a source of food.

### 2.2. Methods

**Figure 1:**
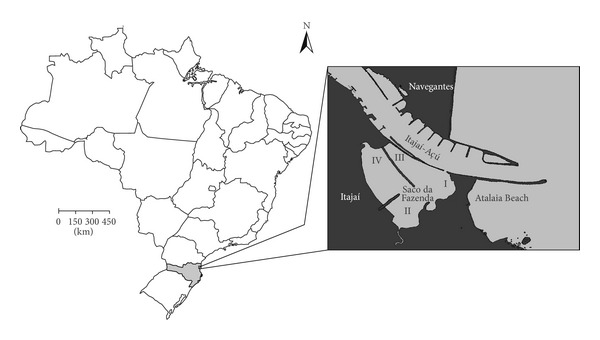
Studied area indicating the collecting sites in Saco da Fazenda, Santa Catarina, Brazil (source: modified of chart 1801 from Diretoria de Hidrografia e Navegação/DHN).

Specimens were collected monthly, in the intertidal and subtidal regions of Saco da Fazenda, in four areas (Figure 1) between July 2003 and June 2004. In each area, six samplings with a Van Veen grab (0.075 m^2^) were conducted during high spring tides. Three samples were obtained in the intertidal region (40 cm depth) and three in the subtidal (140 cm depth). For the analyses the three samples from each region were considered as a single sample (0.225 m^2^ per region).

The tanaidaceans were separated from the sediment with a 0.5 mm mesh-size sieve, fixed in 4% formalin, and preserved in labelled bottles. In the lab, the residual sediment was removed and the total number of specimens per sample was determined.

To estimate the density of males, females, and juveniles along the year, as well as the number of females in reproductive activity, a subsample was established from the total number of collected individuals. To obtain this, captured animals were put in a beaker of 250 mL with water. The material was homogenized and three samples of 20 mL were extracted. We used the mean value of individuals for the three samples and calculated the total number of individuals per sample for that collecting month.

To characterize the structure of the population, we obtained two new samples from the field during the summer and winter of 2005. Due to the low abundance of organisms captured in these samples, the samples were pooled and 220 entire individuals were chosen for the classes of males, females, ovigerous females, and juveniles, which were measured from the tip of the rostrum to the tip of the telson with a micrometric ocular on a stereoscopic microscope [2]. The eggs and mancae in the marsupium of each female were counted; the largest diameter of these was obtained and then classified by developmental stage [11]. The relationship between the length of females and the number of eggs and mancae was determined by simple regression analysis [34], utilizing 80 individuals of each stage, selected randomly among those with intact marsupia.

Temperature and salinity of the bottom water were measured monthly, during the morning and afternoon, from July 2003 to June 2004. The salinity was recorded using a refractometer. A sample of sediment was collected at each area every trimester (four replicates per zone), which was used for granulometric analysis and measurement of calcium carbonate content and organic matter. Calcium carbonate was determined using the gravimetric method, from a sample of 100 g, which was dissolved in hydrochloric acid (HCL) at 50°C. To determine total organic matter, the samples were burned in a muffle furnace at 800°C for eight hours and the content was determined by the difference between initial and final weights. For the granulometric and textural characterization of the sediment, samples were wet-sieved at intervals of 1/4 phi [35] and then weighed [36]. To compare granulometry, calcium carbonate content, and organic matter among stations, between intertidal and subtidal regions, we conducted a one-way ANOVA for each comparison. The data were transformed into the one-fourth root.

To verify if abundance of *M. schubarti* is correlated with temperature and salinity, the Pearson correlation was tested (with data transformed into the one-fourth root). To compare total abundance of *M. schubarti* with the three studied sediment parameters (granulometry, calcium carbonate, and organic matter), we also used one-way ANOVA.

The chi-square test (*χ* ^2^) with Yates correction, at level of significance of 5% (*n* − 1 degrees of freedom), was applied to verify the possible differences among the sex ratio per collecting month and total number of adults.

To evaluate the spatiotemporal variation of tanaidaceans, we applied a two-way ANOVA on data of total abundance and number of individuals for each size class of *M. schubarti* (males, females, ovigerous females, and juveniles) for the different months of the year and regions (intertidal and subtidal). When the data did not fit these requirements, we used the transformation in the fourth root. Such transformations antedated the use of ANOVA, which was followed by Tukey's test (*α* = 0.05).

## 3. Results

### 3.1. Abiotic Parameters

**Figure 2:**
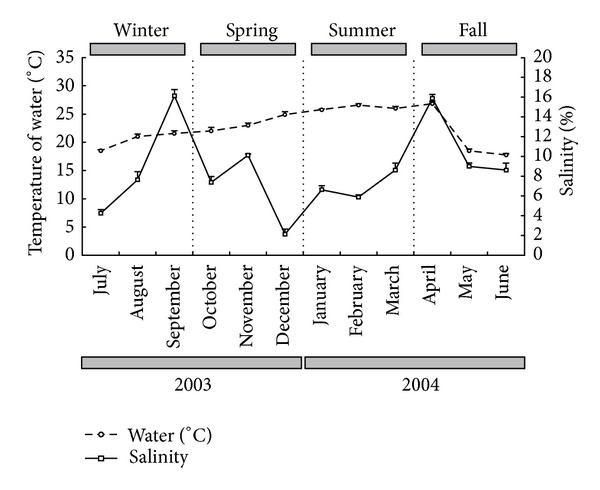
Mean temporal variation of salinity and temperature of bottom water, during the period from July 2003 to June 2004 (mean ± standard error).

The mean bottom water temperature showed a typical seasonal pattern, with significant differences among the studied months (*F* = 69.212; df = 11, 84; *P* < 0.001). Highest mean values occurred in February (26.6°C) and April 2004 (26.8°C) and the smallest occured in May (17.3°C) and June (17.8°C) of this same year (Figure 2).

Salinity varied significantly among collecting months (*F* = 44.043; df = 11, 84; *P* < 0.001). Highest values were measured in September 2003 (16.1) and April 2004 (15.9) and the smallest value was found in December 2003 (2.1) (Figure 2).

Grain size, calcium carbonate content, and organic matter presented significant differences among the intertidal and subtidal regions (*F* = 41.649; *P* < 0.001, *F* = 5.028; *P* = 0.033, *F* = 30.487; *P* < 0.001, resp., df = 1, 30 for all measurements). Mean grain size and mean values of calcium carbonate and organic matter were higher in the subtidal region (8.03 ± 0.23 phi, 7.14 ± 0.58%, 11.05 ± 0.44%, resp.) than in the intertidal region (5.54 ± 0.30 phi, 5.44 ± 0.53%, 6.66 ± 0.62%, resp.).

**Figure 3:**
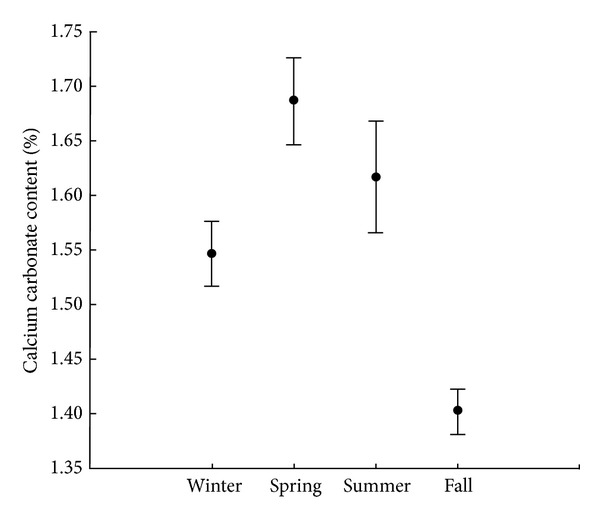
Mean values of calcium carbonate of Saco da Fazenda, during the seasons of sampled period (mean ± standard error).

Calcium carbonate was significantly different when compared among seasons (*F* = 9.653; df = 3, 28; *P* < 0.001) (Figure 3). Fall presented the smallest mean (1.39 ± 0.24%), while the largest mean was observed in spring (1.69 ± 0.78%). Grain size and organic matter content did not vary significantly among stations (*F* = 0.108; *P* = 0.955 and *F* = 0.788; *P* = 0.513, resp., df = 3, 24).

### 3.2. Spatiotemporal Distribution

Total abundance of *M. schubarti* was not correlated to water temperature and salinities in the intertidal regions (*r*_*s* water^ °^C_ = 0.08, *n* = 48, *P* = 0.6; *r*_*s* salinity_ = −0.18, *n* = 48, *P* = 0.2) nor in the subtidal regions (*r*_*s* water^ °^C_ = 0.05, *n* = 48, *P* = 0.7; *r*_*s* salinity_ = −0.09, *n* = 48, *P* = 0.5).

Organic matter and grain size presented a significant negative correlation with the total abundance of *M. schubarti* (*r* = −0.38, *n* = 32, *P* = 0.034; *r* = −0.52, *n* = 32, *P* = 0.003, resp.). The calcium carbonate content did not show a significant correlation with the total abundance of *M. schubarti* (*r* = −0.15, *n* = 32, *P* = 0.404).

**Figure 4:**
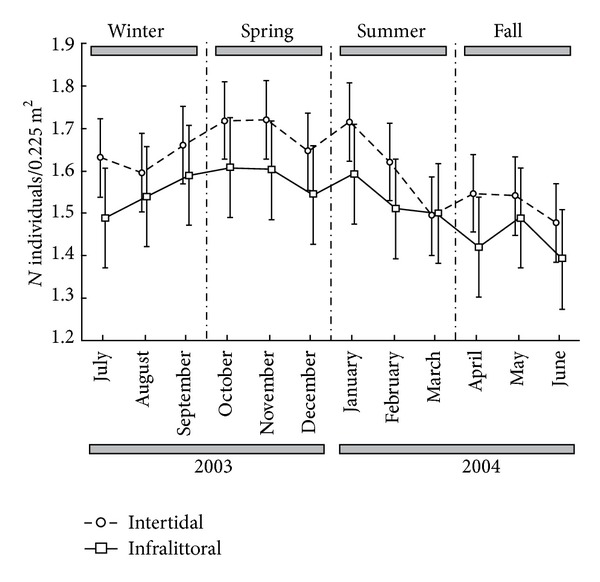
Monthly variation of the mean density of *Monokalliapseudes schubarti *per 0.225 m^2^ (in this case transformed to the fourth root), in intertidal and subtidal of Saco da Fazenda, during the period from July 2003 to June 2004 (mean ± standard error).

Density *M. schubarti* in the intertidal region (Figure 4) was significantly larger than that in the subtidal region (*F* = 1.723; df = 22, 70; *P* = 0.045), while abundances of males, females, ovigerous females, and juveniles were also significantly correlated (*F* = 16.199; *P* < 0.001, *F* = 23.029; *P* < 0.001, *F* = 16.369; *P* < 0.001, *F* = 20.822; *P* < 0.001, resp.; df = 1, 94 for all data).

**Figure 5:**
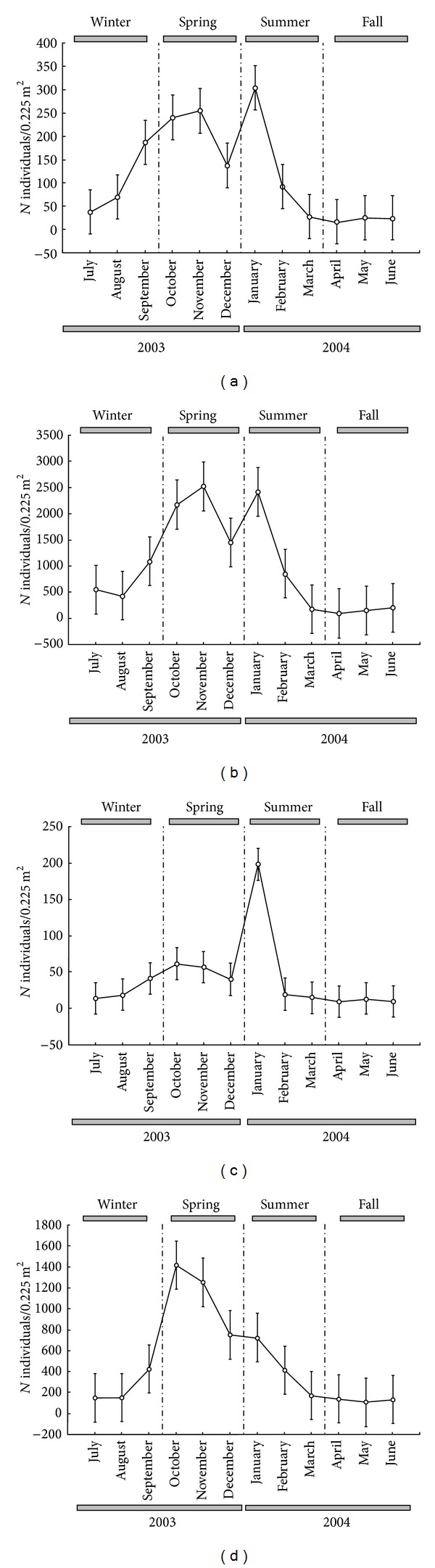
Monthly variation in the mean density of* Monokalliapseudes schubarti *per 0.225 m^2^, of Saco da Fazenda, during the period from July 2003 to June 2004: (a) males; (b) females; (c) ovigerous females; (d) juveniles (mean ± standard error).

 The density of males and females varied monthly (*F* = 4.747 and *F* = 3.895, resp., *P* < 0.001 and df = 11,   84 for both data), with a peak in spring and another in summer, followed by subsequent declines and remaining low during fall (Figures 5(a) and 5(b)).

 Statistical differences were verified in the density of ovigerous females among months (*F* = 5.908; df = 11, 84; *P* < 0.001). However, this density varied only slightly along the year, with the statistical differences being a reflection of the strong peak occurring at the beginning of summer (Figure 5(c)).

Juveniles varied monthly (*F* = 3.919; df = 11, 84; *P* < 0.001), with a peak of density at the beginning of spring, decreasing gradually along the following stations. The lowest values occurred during fall (Figure 5(d)).

### 3.3. Length Structure

The sex ratio (females : males) of the population was 9 : 1. Females strongly predominate (*χ* ^2^ = 8 470.32, *P* < 0.001).

**Figure 6:**
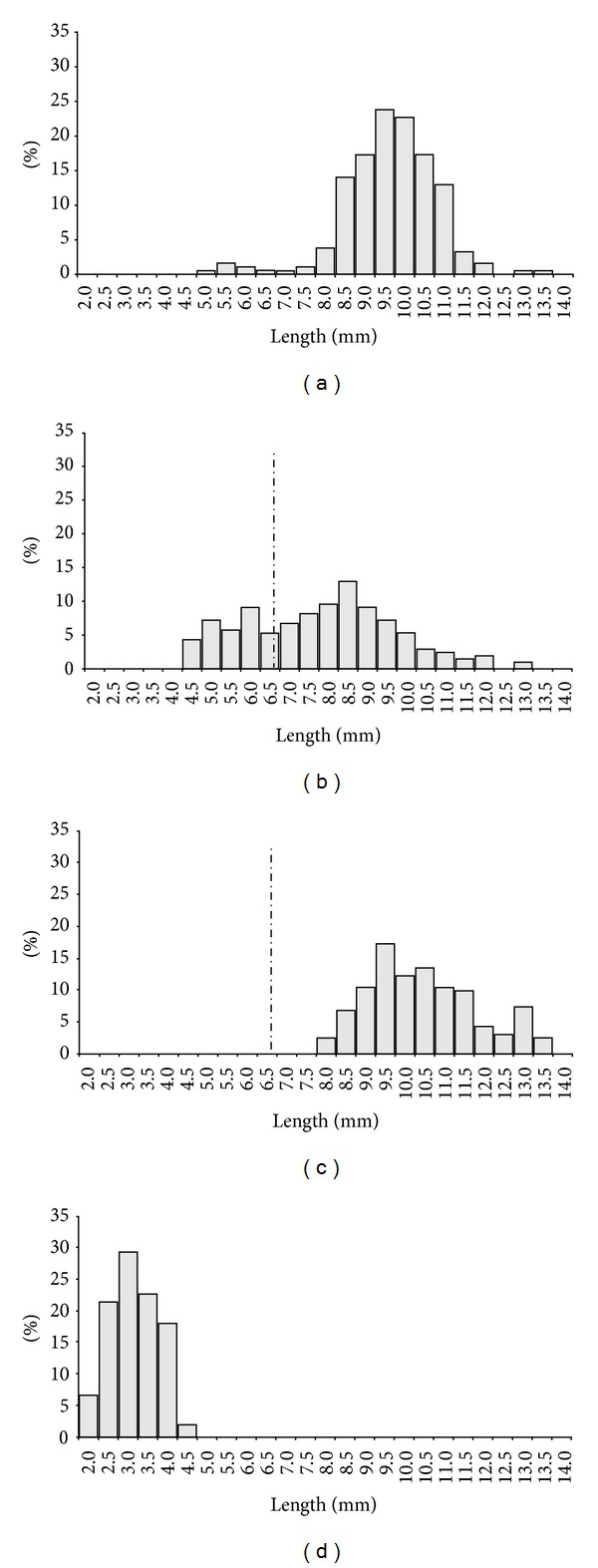
Distribution of frequency by class of length of *Monokalliapseudes schubarti* in Saco da Fazenda, during the period from July 2003 to June 2004: (a) males; (b) females*; (c) ovigerous females*; (d) juveniles (*n* = 220; *traces line = size of first mature stage, according to Fonseca and D'Incao [16]).

The total length for males varied from 5.2 to 13.5 mm, with the largest captures occurring in classes of 9.5 and 10.0 mm and the mean total length being 9.8 ± 0.1 mm (Figure 6(a)).

In females, total length ranged from 4.5 to 13.3 mm, presenting a polymodal distribution, with peaks in the classes of 5.0, 6.0 and 8.5 mm and with a mean length of  7.9 ± 0.1 mm (Figure 6(b)). For ovigerous females, the length varied from 8.2 to 13.9 mm, with modes in the classes of 9.5, 10.5, and 13.0 mm and with a mean size of 10.6 ± 0.1 mm (Figure 6(c)).

In juveniles the range of variation was from 2.0 to 4.5 mm, with a peak in the class of 3.0 mm and a median length of 3.3 ± 0.0 mm (Figure 6(d)).

### 3.4. Reproductive Period and Fecundity

*Monokalliapseudes schubarti* presented females in reproductive activity during the whole sampled period, reinforcing the hypothesis of continuous reproduction. The largest density of ovigerous females was observed in January 2004 (198.25 ± 65.21), while in April 2004 the smallest numbers were registered (9.63 ± 3.36) (Figure 5(c)).

Juveniles were encountered throughout the year. The largest peak of recruitment was registered in October and November 2003 (1415.27 ± 475.58 and 1253.78 ± 332.92, resp.) and the smallest intensity occurred in May 2004, with 107.73 ± 23.73 individuals (Figure 5(d)).

In Table 1 we present the fecundities of *M. schubarti* in relation to their distinct stages and respective sizes.

**Figure 7:**
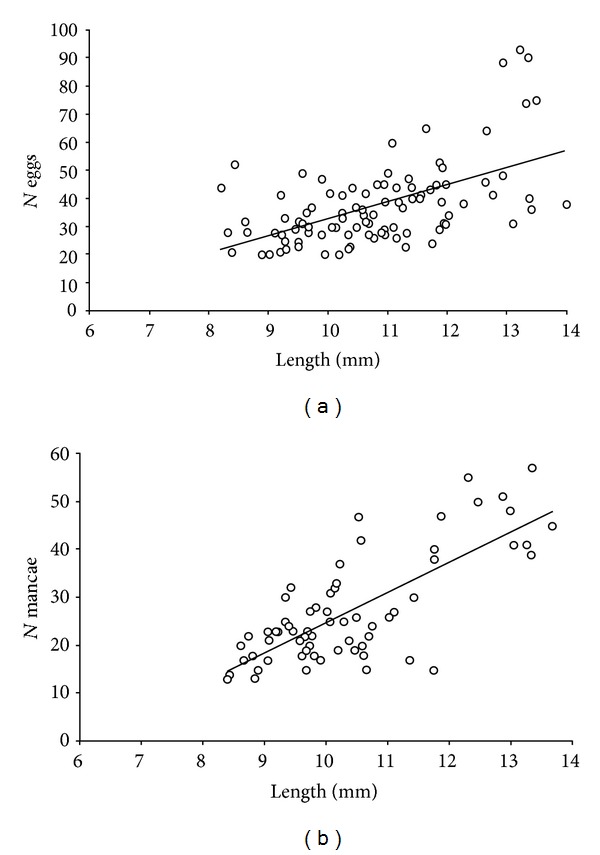
Relationship between the total size of ovigerous females of *Monokalliapseudes schubarti* and the number of: (a) eggs; (b) mancae (*N* = number).

Analyzing the number of eggs and mancae stages relative to the length of females, we verified that fecundity showed a considerable tendency to increase with the size of the female. This correlation was corroborated with the determination coefficients found both for the eggs (*r* ^2^ = 0.31, *P* < 0.001) and for the mancae (*r* ^2^ = 0.57, *P* < 0.001) (Figures 7(a) and 7(b), resp.).

## 4. Discussion

The distribution of* Monokalliapseudes schubarti* is highly aggregated with the greatest densities occurring in estuarine areas and muddy depressions, where they commonly become the dominant species of the macrofauna [4, 14, 21–23, 37].

Several authors have tried to correlate the distribution of this tanaidacean with environmental parameters, in particular, Bemvenuti [4], Leite [13], and Leite et al. [15]. They observed great densities of this species in fine sediment containing much organic matter and a low concentration of calcium. According to them, the organic matter contributes to the availability of food while the carbonates, usually associated with the presence of shells, may interfere with the construction of tubes by the species.

In Saco da Fazenda, on the other hand, the quantity of organic matter was found to be inversely correlated with the abundance of *M. schubarti*. Nucci et al. [38] discussed a preference of *M. schubarti* for high concentrations of organic matter, but their data, obtained for beaches of the São Sebastião Channel, São Paulo, indicate higher densities of this species in localities with low values of organic matter.

Sediment accumulation may interfere with the population of *M. schubarti* from Saco da Fazenda. According to Schettini [39], Saco da Fazenda represents a sedimentary basin for sediments transported by the Itajaí-Açu River, to which it is connected by a single opening. Leite et al. [15] suggest that the density of *M. schubarti* may be influenced by the quantity of silts and clays in the environment, being positive when the quantity is low or intermediate and becoming negative when these sediments attain high values. High concentrations of organic matter may interfere with the development of the species due to the reduction of oxygen, resulting in low densities in these localities.

Regarding temperature and salinity, Leite [13] verified that salinity does not interfere with the distribution of the species, because *M. schubarti* is commonly found in estuarine areas. Brendolan and Soares-Gomes [40] found experimentally that *M. schubarti* is very sensitive when exposed to temperatures above 25°C, which also correlates with the species being restricted to subtropical latitudes.

In the ecosystem of Saco da Fazenda the highest densities occurred in spring and summer. Barreiros et al. [41] observed that in these seasons the abundance of the ichthyofauna was lowest. It thus seems possible that these correlations are functional. According to Freitas-Júnior [42], *M. schubarti* is the main food item of the bony fishes *Centropomus parallelus* and *Micropogonias furnieri*, the dominant fish species in this estuary. Furthermore, *M. schubarti* becomes a common food item not only to fish, but also to crustaceans and birds [3, 4, 13, 20, 41, 43, 44].

The number of females (*n* = 12133) captured in the present study was larger than the number of males (*n* = 1419) over the whole sampled period, resulting in the proportion 9 : 1. Leite et al. [15] found a proportion of 2.69 : 1. This predominance of females may be attributed to the reproductive behavior of the species. Because fertilization occurs inside the tube of the female, males must leave their tubes in search of females becoming more susceptible to predation, desiccation, and dispersal by currents, resulting in a greater differential mortality [15].

Bemvenuti [45] stresses that *M. schubarti* from the estuarine region of Lagoa dos Patos, Rio Grande do Sul, reproduces the whole year round, with peaks of reproductive activity in the most favorable periods of food and temperature. In Saco da Fazenda this species presented females in reproductive activity during the whole sampled time span, reinforcing the hypothesis of continuous reproduction. However, we observed a seasonal pattern of reproduction and recruitment, with larger intensities in spring and summer, and reduced frequencies in fall and winter, corroborating the results of Lana et al. [14], Bemvenuti [5], Leite [13], Leite et al. [15], Fonseca and D'Incao [16], and Colling et al. [46].

The length of the female of *M. schubarti* at sexual maturity in Saco da Fazenda (*L*_50_ = 8.2 mm) was higher than that in Lagoa de Itaipu (*L*_50_ = 5.9 mm) [17] and the Araçá region (*L*_50_ = 5.02 mm) [15]. Maranhão et al. [47] and Nicolau and Oshiro [48] observed that environmental factors and differences in the latitudinal gradient may interfere in the growth and metabolism of individuals, altering the body length at sexual maturity. Thus, females at Saco da Fazenda may be under the influence of latitude.

For Fonseca and D'Incao [16], the size of the first mature stage of the species in Lagoa dos Patos is attained approximately two months after leaving the marsupium (*L*_50_ = 6.6 mm), indicating the high investment that precedes the process of reproduction. According to these authors, the high investment in somatic growth maximizes the reproductive potential of females, permitting that a larger number of mancae stages may be brooded in the marsupium. In Saco da Fazenda, we found a significant correlation between size of females and number of eggs and mancae. This differs from the results in Leite et al. [15], who attributed the weak correlation obtained in their study to the loss of eggs while processing species during collecting. For Fonseca and D'Incao [16], the nonsignificant correlation between female size and number of eggs and mancae is a consequence of the nonsynchronic development of the progeny. They found eggs, embryos, and mancae together in the same marsupium.

The mean number of eggs and mancae observed in our study (Table 1) was higher than that found by Leite et al. [15] (mean values of 11.8 eggs and 7.8 mancae).

In general, *M. schubarti* presented high values of fecundity, with continuous reproductive activity during the whole studied period. Their greatest population densities were observed in the areas that border the ecosystem, where they are intensely consumed by mixed bands of marine and freshwater birds [19]. Furthermore, they constitute an important food item in the diet of swimming crabs of genus *Callinectes* [49] and of gobiid fish [50]. We may consider them a fundamental link in the food chain of Saco da Fazenda, transferring energy from the detritus level to the higher trophic levels.

The predominance of *M. schubarti* along the intertidal region of the estuary may indicate preference for shallow tidal flats or difficulty in colonizing the more highly disturbed river channels of the estuary. On the other hand, the deeper subtidal region, where the smallest population densities of *M. schubarti* were found, also accumulated the highest percentages of organic matter, according to our results. If habitat disturbance and high organic matter are factors controlling the population distribution of *M. schubarti*, we would have two additional factors favoring the use of this species to monitor anthropic effects in estuarine areas.

## Figures and Tables

**Table 1:** Fecundity of *Monokalliapseudes schubarti*, using 80 females per developmental stage.

Embryonic stage	*n*			Length (mm)		
	<	>	Mean ± SE	<	>	Mean ± SE
Egg I	20	93	37 ± 1.81	0.30	0.56	0.45 ± 0.00
Egg II	26	60	36 ± 1.32	0.49	0.82	0.60 ± 0.00
Manca I	15	57	30 ± 1.94	0.70	1.36	1.07 ± 0.01
Manca II	13	51	24 ± 1.79	1.23	2.12	1.63 ± 0.02

*n*: number; <: minimum; >: maximum; SE: standard error.
